# Effects of Mepolizumab in the treatment of type 2 CRSwNP: a real-life clinical study

**DOI:** 10.1007/s00405-024-09027-8

**Published:** 2024-10-15

**Authors:** Pietro Orlando, Emanuele Vivarelli, Alberto Minzoni, Giuseppe Licci, Matteo Accinno, Barbara Brugnoli, Andrea Matucci, Alessandra Vultaggio, Giandomenico Maggiore

**Affiliations:** 1 Department of Otorhinolaryngology, Careggi University Hospital, Largo Brambilla, 3, Florence, 50134 Italy; 2 Immunoallergology Unit, Careggi University Hospital, Florence, Italy; 3 Department of Experimental and Clinical Medicine, University of Florence, Florence, Italy

**Keywords:** CRSwNP, Mepolizumab, Real-life, Sinonasal outcome, Olfactory disfunction

## Abstract

**Purpose:**

Mepolizumab was recently approved for treating Chronic Rhinosinusitis with Nasal Polyps (CRSwNP) unresponsive to standard treatment or recurring after endoscopic sinus surgery (ESS). To date, few studies have assessed Mepolizumab’s efficacy in severe type-2 CRSwNP. Our study aimed to analyze sinonasal outcomes in type-2 CRSwNP patients treated with 100 mg Mepolizumab administered subcutaneously every four weeks.

**Methods:**

We conducted a retrospective study of patients with severe, recalcitrant CRSwNP treated with Mepolizumab. Demographic and clinical characteristics were collected, including age, sex, and comorbidities such as asthma, nonsteroidal anti-inflammatory drug-exacerbated respiratory disease (NERD), and allergic rhinitis (AR), as well as the number of previous ESS procedures and the interval since the last one. Patients were evaluated at baseline and after one year for blood eosinophil count, nasal polyp score (NPS), modified Lund-Kennedy score (mLKS), olfactory function (using a VAS scale and a 16-item Sniffin’ identification test), SNOT-22, and sinus opacification on CT scans. The need for rescue ESS or systemic corticosteroids (SCS), response to treatment, and side effects were also recorded.

**Results:**

Data from 27 patients were collected. After one year, all scores showed significant improvement. NERD was the only factor associated with a less favorable improvement in olfactory function. There were no side effects reported, although 2 patients discontinued Mepolizumab as they were considered “non-responders.”

**Conclusions:**

Mepolizumab is safe and effective in reducing the clinical, endoscopic, and radiological burden of disease, as well as in decreasing the need for salvage ESS or systemic steroids.

## Introduction

Type-2 Chronic Rhinosinusitis with Nasal Polyps (CRSwNP) is a life-limiting condition characterized by severe respiratory nasal blockage and smell impairment, an unsatisfying response to the standard medical treatment, and a high tendency to recur even after endoscopic sinus surgery (ESS). Chronic inflammation is driven by type-2 cytokines, particularly interleukin (IL) -4, IL-5, and IL-13, thus it is often associated with other comorbidities that share the same underlying pathological mechanisms, such as asthma, allergic rhinitis (AR), and atopic dermatitis (AD) [1]. Mepolizumab was recently approved for the treatment of severe and recalcitrant type-2 CRSwNP not adequately controlled with the standard medical and surgical strategies. Mepolizumab is a humanized monoclonal antibody that acts by inhibiting IL-5, a cytokine that plays a pivotal role in the maturation, differentiation, activation, and survival of eosinophils [1, 2]. Previous studies demonstrated Mepolizumab to be safe and effective in decreasing hematic eosinophils, reducing the need for systemic corticosteroids (SCS), and improving clinical outcomes of patients suffering from severe eosinophilic asthma, hypereosinophilic syndrome, and eosinophilic granulomatosis with polyangiitis (EGPA) [3–7]. The phase 3 clinical trial SYNAPSE proved Mepolizumab to be effective in reducing circulating blood eosinophils, nasal polyps dimension, nasal congestion, and the need for rescue systemic corticosteroids (SCC) and ESS in patients affected by type-2 CRSwNP after 52 weeks [8]. Thus, Mepolizumab was recently approved for the treatment of severe and recalcitrant type-2 CRSwNP not adequately controlled with the standard medical and surgical strategies. As of today, only a few real-life studies about the effectiveness of the anti-IL5 in treating CRSwNP have been published. Our study aimed to retrospectively analyze the sinonasal outcomes of patients affected by type-2 CRSwNP and treated with 100 mg Mepolizumab subcutaneously every four weeks.

## Materials and methods

For this retrospective study, we enrolled patients referred to our University Hospital from January 1, 2021, to May 31, 2023, with the following inclusion criteria: age ≥ 18 years; diagnosis of severe Type 2 CRSwNP refractory to standard medical therapy (intranasal corticosteroids); patients who fulfilled the prescription criteria for biological therapy according to the EPOS2020 guidelines; [9] treatment with the monoclonal antibody Mepolizumab (treatment duration ≥ 12 months, dose of 100 mg every 4 weeks). It should be noted that in Italy, Mepolizumab was approved for the treatment of CRSwNP in March 2023 [10]. Therefore, the choice of anti-IL5 therapy prior to this date was based exclusively on clinical features of asthma, according to GINA recommendations.

Type 2 CRSwNP was defined by the presence of atopy or a blood eosinophil count > 250/µL, or ≥ 10 eosinophils/HPF in the histopathological examination of polypoid tissue biopsy, according to the EPOS2020 guidelines [9]. Atopy was defined by a positive clinical history and positive skin testing and/or specific IgE antibodies against common allergens. Demographic, laboratory, and clinical data were retrieved from clinical records. Patients’ demographic characteristics analyzed at baseline included age, sex, smoking habits, and comorbidities such as asthma, nonsteroidal anti-inflammatory drug-exacerbated respiratory disease (NERD), and allergic rhinitis (AR), as well as a previous history and timing of endoscopic sinus surgery (ESS). Blood eosinophil count and sinonasal-related clinical data were collected at baseline (before Mepolizumab prescription) and 12 months after starting Mepolizumab.

Sinonasal-related quality of life was assessed using the SNOT-22 score [11]. The endoscopic severity of CRSwNP was evaluated with two different staging systems during the videoendoscopic examination of the nasal fossae: the Meltzer NPS [12] and the modified Lund-Kennedy score (mLKS) [13]. The NPS was used to assess the size of nasal polyps in relation to the middle turbinate, according to the Meltzer staging system, while the mLKS was used to evaluate nasal polyp size, the severity of nasal edema, and the quality and quantity of nasal secretions. The sense of smell was assessed using an 11-point (0–10) visual analogue scale (VAS) for subjective olfactory dysfunction and the 16-SSIT score [ODOFIN, Burghart Messtechnik GmbH, Holm, Germany ©]: a score ≥ 12/16 was considered normosmia, while a score ≤ 7/16 was considered anosmia; intermediate scores (8–11) were classified as hyposmia [14]. A CT scan was used to assess the opacification of nasal fossae and paranasal sinuses based on the Lund-Mackay score (LMS) [15]. Asthma-related outcomes were evaluated using the asthma control test (ACT) and the asthma control questionnaire (ACQ-5) [16, 17]. Finally, the need for SCS or ESS over the study period was collected, as well as the onset of adverse events (AEs).

For statistical analysis, a two-tailed t-test and the Mann-Whitney test were used for unpaired data, while the paired t-test and the Wilcoxon signed-rank test were used for paired data, depending on the data distribution. The Shapiro-Wilk test was used to check for the normality of data. Descriptive and statistical analyses were performed using SPSS software (IBM Corp. Released 2020. IBM SPSS Statistics for Macintosh, Version 27.0. Armonk, NY: IBM Corp). All statistical tests were two-sided, and a p-value of less than 0.05 was considered statistically significant. Approval from our Local Ethics Committee was obtained.

## Results

### Study population

A patient cohort of 27 patients was enrolled in the present study. In our population, males and female subjects were equally represented (51.9% vs. 48.1%); there was little history of smoking exposure (3.7%) and mean age at enrollment was 57.7 years old (yo) (range 27–79 yo). Baseline blood eosinophils were quite high 800 [Interquantile range (IQR) 500.00–1060.00 cells/µL]. All baseline features are shown in Table 1.

**Table 1:** Patients’ demographic characteristics at baseline

		Frequency	Percent
Sex	male	14	51.9
	female	13	48.1
*Smoking history*	*no*	*26*	*96.3*
	*yes*	*1*	*3.7*
*Allergic Rhinitis*	*no*	*13*	*48.1*
	*yes*	*14*	*51.9*
*Asthma*	*no*	*2*	*7.4*
	*mild*	*4*	*14.8*
	*severe*	*21*	*77.8*
*NERD*	*no*	*17*	*63.0*
	*yes*	*10*	*37.0*
*previous ESS*	*no*	*8*	*29.6*
	*yes*	*19*	*70.4*
	*Total*	*27*	*100*

Acronyms: N-ERD, nonsteroidal antiinflammatory drugs-exacerbated respiratory disease; ESS, endoscopic sinus surgery

19 patients (70.4%) began treatment before March 2023, using asthma criteria according to GINA recommendations, and subsequently meeting EPOS criteria for inclusion in the study.

Concerning relevant comorbidities, 25/27(92.6%) patients were affected by bronchial asthma (BA), mostly severe BA (21/27, 77.8%), and a significant proportion were atopic, as shown by the presence of allergic rhinitis (14/27, 51.9%); a minority of patients was affected by non-erosive gastroesophageal reflux (NERD). Moreover, most patients had undergone endoscopic sinonasal surgery (ESS; 19/27, 70.4%) with a mean ESS to Mepolizumab treatment time of 106 months (range 12–198, standard deviation 60.53).

Baseline sinonasal scores demonstrated a severe impairment of nasal anatomy and physiology, as well as of quality of life (SNOT-22 58.56 [IQR 45.00-69.50]; NPS 5.56 [IQR 5.00–6.00]; mLKS 6.59 [ IQR6.00–8.00]; SSIT 4.96 [IQR 4.00–6.00]; Loss of smell VAS 9.56 [IQR 10.00–10.00]; Lund-Mackay score 12.81 [IQR 8.00–16.75] ). Baseline asthma scores were suggestive of uncontrolled asthma (ACT 17.00 [IQR15.00–19.00]; ACQ-5 2.23 [IQR 1.30–2.80]).

### Mepolizumab is effective in improving both sinonasal scores and quality of life

**Fig. 1:**
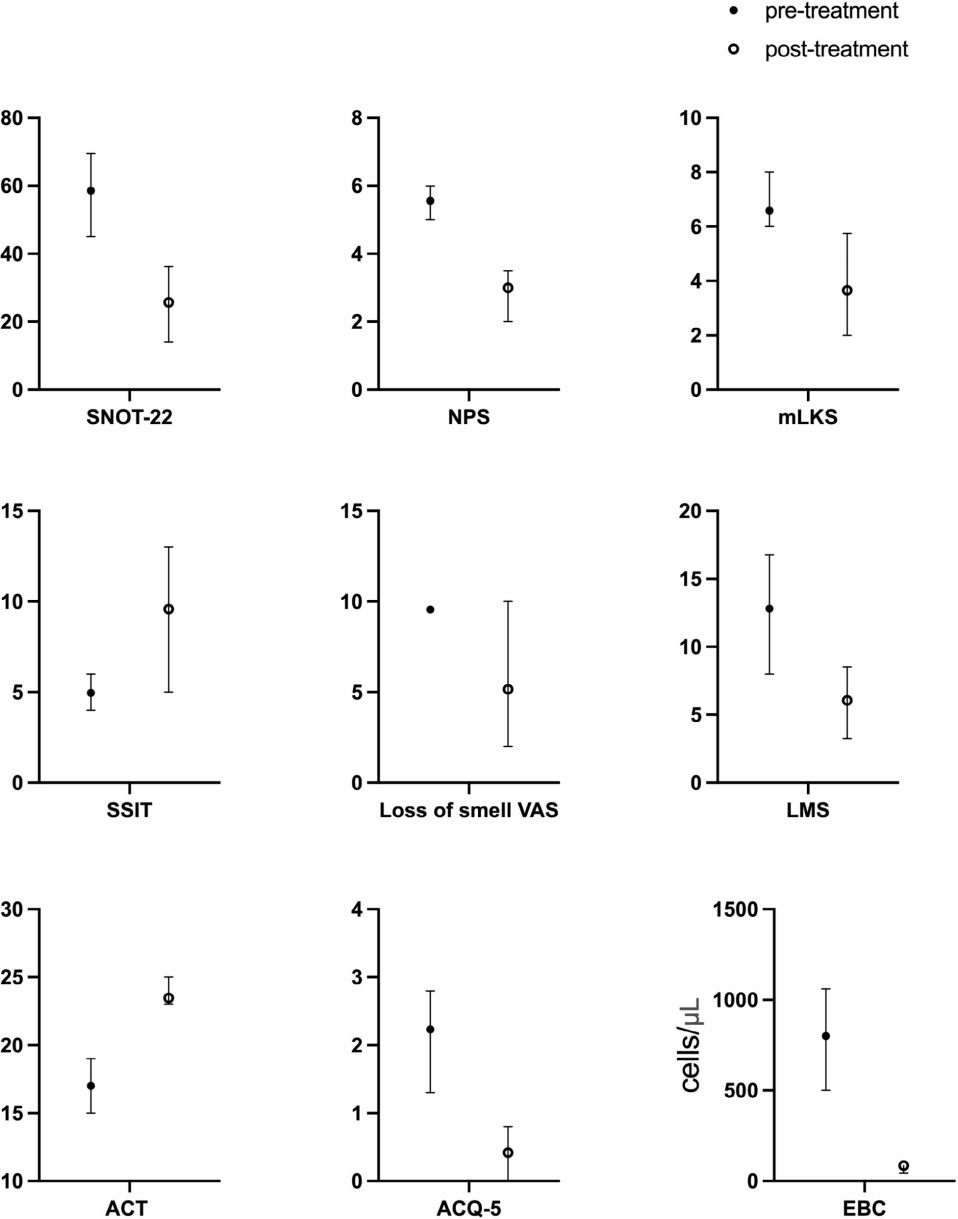
Box plot with mean scores and 1st and 3rd interquartiles pre and post-treatment for the study scores and the parameters. *Acronyms: SNOT-22*,* SinoNasal Outcome Test-22; NPS*,* Nasal Polyp Score; mLKS*,* modified Lund-Kennedy Score; LMS*,* Lund-Mackay score; SSIT*,* Sniffin’ sticks identification test*,* ACT*,* asthma control test (only for asthmatic patient); ACQ-5*,* asthma control questionnaire-5 (only for asthmatic patient)*

After one year of Mepolizumab treatment, our study cohort demonstrated significant improvement in all sinonasal scores (see Fig. 1 ).

Specifically, SNOT-22 significantly improved (58.6 vs. 25.7, with 26 vs. 27 data points, *p* < 0.005), with almost all patients achieving the minimal clinically significant variation of at least 8.9 points (25/27, 92.6%, see Table 2).

**Table 2:** Effectiveness evaluation of Mepolizumab in reduction of scores

		Frequency	percent
**reduced NPS**	no	5	18.53
	yes	22	81.5
**reduced OCS**	no	2	7.4
	yes	25	92.6
**Reduced SNOT-22**	no	0	0.0
	yes	27	100.0
**Reduced impact of comorbidities**	no	1	4.0
	yes	24	96.0
**Improved smell (SSIT)**	no	6	22.2
	yes	21	77.8
**CRSwNP control**	poor	2	7.4
	moderate	10	37.0
	excellent	15	55.6
**reduced NPS ≥ 2**	no	8	30.8
	yes	18	69.2
**MCID SNOT22 ≥ 8.9**	no	2	7.4
	yes	25	92.6

*acronyms: NPS*,* nasal polyp score; OCS: oral corticosteroids; SNOT-22*,* SinoNasal Outcome Test-22; MCID: minimal clinically important difference*

NPS significantly declined from 5.6 to 3 (27 vs. 27 data points, *p* < 0.001) as well as mLKS (6.6 vs. 3.6, 26 vs. 27 data points, *p* < 0.001) and Lund-Mackay score (12.8 vs. 6.5, 27 vs. 12 data points *p* < 0.05). Most patients showed a high reduction (≥ 2 points) of NPS (18/27, 69.2%).

We also observed a significant gain in the sense of smell, a very bothersome symptom in CRSwNP, both from an objective (SSIT from 5 to 9.6, 23 vs. 27 data points, *p* < 0.001) and a subjective (Loss of smell VAS from 9.6 to 5.2, 26 vs. 27 data points, *p* < 0.001) point of view. Anosmic patients were significantly reduced (from 22/27(81.5%) to 12/27(44.4%), *p* = 0.005).

As expected, blood eosinophils significantly decreased (800.00 vs. 85.23, 27 vs. 27 data points, *p* < 0.001) and asthma control significantly improved (ACT 17 vs. 23.5, 25 vs. 25 data points, *p* < 0.001; ACQ5 2.2 vs. 0.4, 25 vs. 25 data points, *p* < 0.001).

### Subgroup analysis of response to Mepolizumab treatment and safety concerns

To deepen our understanding of the response to the biological drug treatment, we performed a subgroup analysis to look for variables influencing Mepolizumab effectiveness in our study population (see Table 3).

**Table 3:** Mean changes (Δ) in scores between subgroups before and after treatment, with the significance of the statistical analysis of their difference. A negative value indicates a decrease in the score. Numbers in parentheses indicate the number of available data points. * denotes a p-value < 0.05

	Asthma			AR			NERD			PREVIOUS ESS		
	Mild (*n*)	Severe (*n*)	*p*	No (*n*)	Yes (*n*)	*p*	No (*n*)	Yes (*n*)	*p*	No (*n*)	Yes (*n*)	*p*
**ΔSNOT-22**	-39.67 (3)	-30.90 (21)	0.630	-34.33 (12)	-29.79 (14)	0.246	-34.12 (17)	-27.67 (9)	0.396	-30.50 (7)	-33.07 (19)	0.494
**ΔNPS**	-2.59 (4)	-2.52 (21)	0.939	-3.07 (13)	-2.07 (14)	0.161	-2.29 (17)	-3.00 (10)	0.244	-2.46 (8)	-3.64 (19)	0.787
**ΔmLKS**	-2.33 (3)	-3.10 (21)	0.301	-3.50 (12)	-3.0 (14)	0.586	-2.76 (17)	-4.11 (9)	0.154	-3.17 (7)	-3.29 (19)	0.187
**ΔSSIT**	-5.34 (4)	-4.38 (18)	0.613	3.56 (10)	8.16 (13)	0.712	4.93 (14)	2.00 (9)	0.043*	5.27 (7)	2.45 (16)	0.339
**ΔSmell VAS**	4.33 (4)	3.50 (21)	0.451	-4.34 (12)	-4.0 (14)	0.821	-5.64 (17)	-3.4 (9)	0.161	-5.6 (7)	-4.14 (19)	0.054
**ΔACT**	4.75 (4)	6.39 (21)	0.423	6.23 (12)	6.15 (13)	0.957	6.18 (16)	6.22 (9)	0.976	6.32 (8)	6.08 (17)	0.871
**ΔACQ-5**	-1.23 (4)	-2.16 (21)	0.180	-2.14 (12)	-1.89 (13)	0.630	-2.04 (16)	-1.98 (9)	0.908	-1.85 (8)	-2.18 (17)	0.519
**ΔBEC**	-960 (4)	-611.84 (21)	0.099	-664.1 (13)	-714.6 (14)	0.870	-723.21 (17)	-630.00 (10)	0.815	-666.50 (8)	-708.33 (19)	0.877

*Acronyms*: *AR: allergic rhinitis*,* NERD: nonsteroidal antiinflammatory drugs-exacerbated respiratory disease; ESS: endoscopic sinus surgery; SNOT-22*,* SinoNasal Outcome Test-22; NPS*,* nasal polyp score; mLKS*,* modified Lund-Kennedy score; SSIT*,* Sniffin’ sticks identification test; LMS*,* Lund-Mackay score; BEC*,* blood eosinophils count; ACT*,* asthma control test (only for asthmatic patient); ACQ-5*,* ashtma control questionnaire-5 (only for asthmatic patient)*

We did not observe significant variations in the response to Mepolizumab according to sex, age, smoking exposure, comorbid asthma or atopy. However, NERD was associated with a significantly reduced gain in smell after treatment (ΔSSIT 2.00 vs. 4.9, *p* < 0.05). As for surgery, there are no differences in scores between patients with prior ESS and surgery-naive patients, although we observed, albeit not statistically significant, that patients who started Mepolizumab within 12 months of surgery had better performance in the scores (ΔNPS − 3.0 vs-2.38; ΔmLKS − 6.0 vs. -3.08; ΔSNOT-22 -31.0 vs. -33.23; Δ Loss of smell VAS − 8.0 vs. -3.846; all *p* > 0.05).

No relevant safety concerns arose during the treatment period and no rescue ESS was performed in our study cohort. However, two patients (7.4%) required SCS and discontinued Mepolizumab as they were considered non-responders: the former did not experience any improvement in asthma symptoms, so she was switched to Benralizumab; the latter did not show any improvement in sinonasal symptomatology, so he was switched to Dupilumab.

## Discussion

Type-2 CRSwNP has historically been defined as a difficult-to-treat disease with a limited response to standard medical treatment and a high recurrence of nasal polyps even after full ESS [18]. Type-2 inflammation is mainly driven by IL-4, IL-5, and IL-13. Specifically, IL-5 promotes bone marrow release, recruitment, and tissue survival of eosinophils in type-2 CRSwNP [19]. Mepolizumab acts systemically by inhibiting the IL-5 pathway, thus reducing eosinophil production in the bone marrow and their recruitment and activation into the tissue [20]. Recent studies have shown that Mepolizumab may reduce peripheral blood inflammatory eosinophils (iEOS) in patients affected by CRSwNP and asthma [21]. Consistently, we highlighted a substantial reduction in the size of nasal polyps, mucus secretion, mucosal edema, and sinuses opacification, as demonstrated by the statistically significant improvement of NPS, mLKS, and LMS in patients suffering from CRSwNP and treated with Mepolizumab for 52 weeks [8]. Findings from our case series are in line with the SYNAPSE trial and other recently published real-life studies that reported promising results. In fact, real life studies documented a significant improvement in quality of life, respectively assessed by SNOT-22 or Rhinosinusitis Outcomes Measure-31 (RSOM-31) after 6 and 12 months of treatment with the anti-IL5 [22–26]. Furthermore, it has been reported a significant reduction of NPS and loss of smell VAS at the end of the study period [23–25]. Barroso documented a partial (from anosmia to hyposmia) or a total (from anosmia to normosmia) gain in smell function in 22% and 14% of enrolled subjects, respectively, with the best outcomes obtained by patients with atopy, larger nasal polyp size at baseline, and concomitant short cycles of SCS [27]. Interestingly, only comorbid NERD was found to be related to a lower gain in smell function at 16-SSIT in our cohort. Dominguez-Sosa et al. reported higher variations in NPS and hematic eosinophil count among patients affected by NERD compared to non-NERD patients [26]. The nasal tissue of NERD patients has been demonstrated to have a high expression of IL-5R𝛂, supporting the biological activity of IL-5 beyond eosinophils and severe sinonasal inflammation [28]. This may imply a longstanding loss of smell, potentially implicating structural and functional brain reorganization in the olfactory areas [29–31]. Surprisingly, we did not find any difference in outcomes with regard to prior ESS. On the contrary, Dominguez-Sosa previously documented a worse smell gain in patients with a greater number of past ESS [26]. However, we observed that patients with a short interval between the last ESS and the beginning of the anti-IL5 regimen achieved better outcomes compared to patients with a post-surgery period > 12 months, highlighting a possible synergic role for surgery and biologic. We may speculate that prompt initiation of biologic therapy may mitigate post-surgical iatrogenic fibrosis and nervous fiber damage, thus preserving the integrity of the sensorineural transmission pathway [32]. A lower concentration of eosinophils in the olfactory neuroepithelium may diminish the edema and direct neurotoxic effect of certain inflammatory mediators, regardless of olfactory cleft obstruction [20–33]. 

Finally, Mepolizumab was demonstrated to be well-tolerated, as we did not collect any AEs during the study period. In other real-life studies AEs have been complained of by 24 out of 99 patients (24%), with only 2 of them interrupting treatment due to severe AEs (arthritis and gastroenteritis) [22–25]. In the SYNAPSE study AEs were reported by 30 patients (15%). However, 2 patients from our cohort had to discontinue Mepolizumab because they were considered “non-responders” (7.4%), with only one of them not achieving satisfactory clinical control of CRSwNP (3.7%). Our findings are in line with those reported by other real-life studies and SYNAPSE trial with the 8.7% of patients with neither SCS or ESS by week 52 who were considered as “non-responders” [8]. 

The small number of patients, the short follow-up period, and the high percentage of asthma patients certainly represented the major limitations of our study. The high percentage of asthmatic patients is not solely due to those who began treatment before March 2023: of the 8 patients treated after this date, only 2 (25.0%) are non-asthmatic, and all exhibit high pre-treatment eosinophil values (mean EBC 816.25; IQR 510–1145). Additionally, we assessed smell function only with an identification test, lacking a threshold and a discrimination olfactory test. Hence, no solid conclusions may be drawn about the effectiveness and safety of Mepolizumab in treating CRSwNP patients. We recognize that universally accepted guidelines for the choice of the right monoclonal antibody in type-2 CRSwNP patients are still lacking. Additionally, no clinical biomarkers or clinical factors have been identified as predictors of response to biologics. The high safety profile and the capacity to reduce circulating eosinophils made Mepolizumab the best option for patients with type-2 CRSwNP, severe asthma, unfit for surgery, and concomitant heavy levels of hematic eosinophils (> 1500 cell/µL), both at baseline or arose after anti-IL-4/IL-13 administration [34, 35]. 

To the best of our knowledge, this is the first real-life study considering two different endoscopic scores, evaluating the sense of smell through a VAS and a 16-SSIT, and analyzing the radiological changes of sinonasal cavities in patients affected by severe and recalcitrant type-2 CRSwNP and treated with Mepolizumab.

## Conclusion

The anti-IL5 Mepolizumab represents a therapeutic option for patients affected by severe type-2 CRSwNP and in particular in those with high levels of circulating eosinophils, as it was proven to be safe and effective in improving hematic eosinophils count, sinonasal-related quality of life, sense of smell, endoscopic scores, and sinonasal opacification at the CT scan after one year, regardless from demographic and anamnestic factors. More studies with larger cohorts and longer follow-up periods are needed to reveal the real impact of Mepolizumab, its possible long-term complications, and its interaction with surgery.

## Data Availability

All data supporting the findings of this study are available from the corresponding author upon reasonable request.
